# Older Adults Experiences of Learning to Use Tablet Computers: A Mixed Methods Study

**DOI:** 10.3389/fpsyg.2018.01631

**Published:** 2018-09-03

**Authors:** Eleftheria Vaportzis, Maria Giatsi Clausen, Alan J. Gow

**Affiliations:** ^1^Department of Psychology, School of Social Sciences, Heriot-Watt University, Edinburgh, United Kingdom; ^2^Division of Occupational Therapy and Arts Therapies, School of Health Sciences, Queen Margaret University, Edinburgh, United Kingdom; ^3^The Centre for Person-centred Practice Research, Queen Margaret University, Edinburgh, United Kingdom; ^4^Centre for Cognitive Ageing and Cognitive Epidemiology, University of Edinburgh, Edinburgh, United Kingdom

**Keywords:** aging, older adults, tablet computers, technology, focus groups

## Abstract

**Background:** We wanted to understand older adults’ experiences of learning how to use a tablet computer in the context of an intervention trial, including what they found helpful or unhelpful about the tablet training, to guide future intervention studies.

**Methods:** Mixed methods study using questionnaire and focus group approaches. Forty-three participants aged between 65 and 76 years old from the “Tablet for Healthy Ageing” study (comprising 22 in the intervention group and 21 controls) completed a post-intervention tablet experience questionnaire. Those who completed the tablet training intervention were invited to share their experiences of engaging with new technology in post-intervention focus groups. We conducted three separate focus groups with 14 healthy older adults (10 females).

**Results:** Questionnaire data suggested that the overall experience of the 22 participants who participated in the tablet training intervention was positive. The majority of participants said that it was likely or very likely they would use a tablet in the future. The focus group themes that emerged were related to the perception of tablet training, the experience of using tablets, and suggestions for future studies. Participants mentioned that their confidence was increased, that they enjoyed being part of a social group and downloading applications, but they also felt challenged at times. Advantages of using tablets included the ability to keep in touch with family and friends, a motivation to contribute to the community, and the potential for tablets to improve mental abilities and overall health and wellbeing. Participants made suggestions that would enable tablet usage, including improvement of features, and suggestions that would improve future tablet training studies, including smaller classes.

**Conclusion:** Our findings have implications for the development of interventions utilizing new technologies that might promote the health and wellbeing of older adults.

## Introduction

Mobile technological devices such as tablet computers (commonly referred to as tablets), a type of portable computer that has a touchscreen, continue to rise in popularity. Tablets were first introduced in 1987, however, it was not until the release of the iPad in 2010 that they gained momentum (Danver, 2016). In recent years, the number of adults aged 65–74 years using tablets in the United Kingdom increased from 39% in 2015 to 51% in 2016 (Ofcom, 2017a). Tablets have the potential to improve older people’s quality of life by facilitating independent living (Orpwood et al., 2010). They often offer the same functionality as a normal computer at a smaller size and lighter weight. Tablets can also assist in bridging the technological gap across generations by teaching them to use technological devices (Bailey and Ngwenyama, 2010). According to Ofcom (2017b), there has been a sharp increase in over-75s using tablets, from 15% in 2015 to 27% in 2016. Older adults may prefer tablet technology over traditional computer technology due to the portability and usability advantages (e.g., adjustable font or icon size), especially those older people experiencing a wide range of specific motor and visual requirements (Chan et al., 2016).

### Interventions Using Tablets

Tablets may provide timely interventions to assist older adults in keeping healthy and independent for longer (Chan et al., 2016). Engaging in cognitively demanding tasks that require new learning (e.g., tablet training) has been associated with maintenance of cognitive abilities. The use-it-or-lose it theory proposes that increases in cognitive activity have the potential to reduce cognitive decline associated with healthy and pathological aging (Park et al., 2013). Participating in activities that involve new learning experiences and acquiring new skills may train a number of cognitive abilities such as memory and executive function (Heinz et al., 2013). Therefore, cognitive engagement may offer opportunities to produce broader benefits that are transferable to real-life activities, and new technologies may provide a relevant source of such challenging learning experiences for older adults.

We previously conducted a tablet intervention study, a “Tablet for Healthy Ageing,” in which 22 participants with minimal or no tablet experience completed a 10-week tablet training course (a further 21 participants were included in a control group that received no tablet training) (Vaportzis et al., 2017b). Intervention participants attended one class per week, and each class consisted of a 2-h instructor-led session. In addition, intervention participants completed homework activities in their own time; over the 10-week program, the average time engaging with the tablet including both in-class and homework/personal usage was 94 h. The 1st week of classes focused on learning the functions of the tablet (e.g., settings, charging) and discovering the variety of applications available. Following weeks were organized by theme. For example, for one theme, “Traveling,” participants learned how to navigate, find travel apps and local resources apps. For another theme, “Entertainment,” they learned how to access music, movies, health and fitness apps, YouTube, etc.

Immediately pre- and post-intervention, we collected and analyzed data on cognitive, health and wellbeing outcomes to provide results on the efficacy of the intervention. Briefly, participants who engaged in the tablet training experienced improvements in processing speed versus participants in the control group (Vaportzis et al., 2017b). Participants who received tablet training were subsequently invited to take part in post-intervention focus groups to explore their tablet training experience in greater depth, to understand what they found helpful or unhelpful about the tablet training, and to guide improvement to future intervention studies.

Our intervention followed from Chan et al. (2016) who trained 18 older computer novices (60–90 years) to use tablets. Participants attended a tablet training course once a week for 3 months. Cognitive performance was compared to a placebo group that engaged in passive tasks requiring limited new learning, and a social group that had regular social interaction but no active skill acquisition. The tablet group showed improvements in episodic memory and processing speed compared with both control groups. These and our results suggest that a brief tablet training intervention had beneficial effects on some cognitive functions in older adults.

We note that prior to the tablet intervention study, we conducted focus groups with a different sample of older adults with no prior tablet computer use and have reported their attitudes to new technology in general and specifically tablets (Vaportzis et al., 2017a). Specifically, 18 healthy older adults between 65 and 76 years (*M* = 71.1, *SD* = 3.7), who were novice tablet users, participated in discussions about the perceived advantages and disadvantages of using tablets, familiarity with, and barriers to interacting with tablets. Participants were also given hands-on experience with various tablet models for the first time. Overall, participants enjoyed the tablet experience, and emphasized the likelihood of using a tablet in the future (Vaportzis et al., 2017a).

**FIGURE 1:**
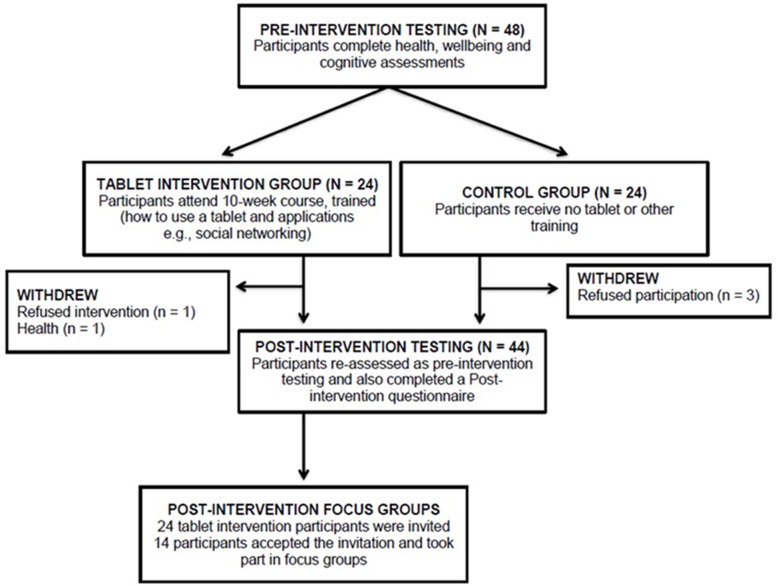
Consort diagram.

Following the pre-intervention focus groups, we conducted the tablet intervention study and reported the effect of tablet training on cognitive ability in a sample recruited for that purpose (Vaportzis et al., 2017b). The current paper reports on these participants’ post-intervention attitudes to their experience from the tablet training intervention study. **Figure 1** shows how the different groups were created from the initial sample.

### Attitudes to and Perceptions of Learning New Technologies

Over the past couple of decades, conflicting results have been found about older adults’ attitudes toward new technologies. In line with an assumption held by the general population (Czaja and Sharit, 1998), research has suggested that older adults’ experiences and attitudes are negative, especially in comparison with younger generations (Timmermann, 1998).

Other studies have, however, contended that older adults’ showed positive attitudes toward new technologies (González et al., 2012). A report by the American Association of Retired Persons (AARP) also showed that although older adults have lower awareness of new technologies, they are generally positive about using them (Barrett, 2011).

Heinz et al. (2013) found that older adults were willing to adopt new technologies when their usefulness and usability surpassed feelings of inadequacy. Despite that, participants’ concerns remained over society’s overreliance on technology, the complexity of technological devices, and a loss of social contact. Mitzner et al. (2010) found that positive reactions to technology included portability and communication, whereas disadvantages included too many options and unsolicited communication.

Perceptions about one’s own ability to learn new technologies have also been explored. Typically, people decide to undertake activities according to their perception of their competence and their ability to complete an activity successfully. When dealing with technologies, older adults often feel unable to use them adequately. This negative belief may be linked to a lower willingness to learn and use new technologies, and even to poor performance when using them (González et al., 2012). More positive perceptions may lead to willingness to learn and use technology, and result to good performance; for example, Gatti et al. (2017) presented a training program that increased self-efficiency in older adults and enabled the learning perception and use of tablets. Therefore, under appropriate instruction and guidance, older adults’ perceptions toward new technologies may change. A number of theoretical frameworks have also been proposed to explain technology acceptance and adoption (e.g., Davis et al., 1989; Venkatesh et al., 2003). The Technology Acceptance Model (TAM; Davis, 1989; Davis et al., 1989) and its successors (Venkatesh and Davis, 2000; Venkatesh et al., 2003) posit that the perceived usefulness and perceived ease-of-use of technology influence how users accept new technology. People are more likely adopt a new piece of technology if they believe it will enhance their performance and if that is effortless (Davis, 1989). Given this, it is therefore necessary to more fully understand the ways in which older people are likely to engage with new technology if those are to be proposed as mechanisms through which interventions might be delivered in the future.

Findings on the use of technology among community-dwelling adults have also been reported by the Center for Research and Education on Aging and Technology Enhancement (CREATE). They found that older adults (60–91 years) were less likely than younger adults to use technology in general, and specifically computers and the internet. Technology adoption was associated with higher cognitive ability, computer self-efficacy and lower computer anxiety, whereas higher fluid intelligence and crystallized intelligence predicted technology adoption; higher computer anxiety predicted lower technology adoption (Czaja et al., 2006). Another study suggested adults aged 60–75 years old perceived less comfort, efficacy and control over computers relative to younger participants. Nevertheless, direct experience with computers resulted in more positive attitudes (Czaja and Sharit, 1998). Overall, the current literature suggests that older adults are open to using technology, but there may be age-related (e.g., cognitive decline) and technology-related (e.g., interface usability) barriers.

As discussed, new technologies including tablets have been increasingly used in interventions with older adults (Zaccarelli et al., 2013; Delbaere et al., 2015; Chan et al., 2016; Taha et al., 2016; Vaportzis et al., 2017b). Although the general focus is on the efficacy of interventions, qualitative data may provide valuable insights on the reasons behind an intervention’s success or otherwise, including the potential barriers to adherence. Despite that, most studies fail to provide an in-depth account post-intervention. To our knowledge, this is the first study to conduct focus groups following a tablet intervention with older adults. The overall aim of the current study was to gain insights relating to older adults’ participation in the “Tablet for Healthy Ageing” intervention program to understand what they found helpful or unhelpful about the tablet training, and to guide future research. The study addressed a number of questions relevant to implementation and adherence, and the outcomes could improve protocols when designing future studies. We employed a mixed methods approach using a post-intervention tablet experience questionnaire, as well as focus groups which provide an open and exploratory approach for qualitative data collection (Krueger, 1998). We wanted to understand: (a) the aspects of the tablet training course that participants liked and did not like; (b) whether or not a tablet was perceived as helpful in assisting with everyday living; and (c) whether or not participants felt that the tablet improved their mental abilities, general health and wellbeing. Finally, we wanted to collect information to assist in the design future intervention studies.

## Materials and Methods

### Participants

Forty-three community-dwelling older adults between the ages of 65 and 76 (*M* = 69.1, *SD* = 3.3) who participated in the “Tablet for Healthy Ageing” study completed a post-intervention tablet experience questionnaire. There were 22 participants in the tablet training intervention group, and 21 participants in a no-contact control group. Participants were randomly assigned to the intervention (i.e., tablet training) or control group. A computerized block randomization procedure was implemented using www.sealedenvelope.com with a block size of four or six participants. Randomization was stratified by sex. Group allocation was disclosed to participants only after all participants completed the pre-intervention testing. Participants in the no-contact group were asked not to start tablet training or engage with tablets until they completed the post-intervention training; participants were asked to verify this upon their second visit. Of the 22 participants in the tablet training group, 14 accepted an invitation to take part in post-intervention focus groups [aged 65–75 (*M* = 68.2; *SD* = 3.2)].

Focus group size was based on previous literature, though the ideal size of groups, or optimal participant-instructor ratio, is debated. Arbaugh and Benbunan-Fich (2004) suggest that the group size-outcome relationship is curvilinear; a certain number of participants is necessary for the groups to run, however, a group size beyond this specific number of participants could impede the discussion and make it difficult to facilitate [increasing group size might facilitate discussions to a point but adding participants could also reduce the depth of interaction and the ease of managing the group (Kenny, 2005)]. Khan et al. (1991) propose groups of 4–6 respondents suggesting that the themes generated do not necessarily increase as group size increases. We therefore opted for a combination of focus group sizes.

The 14 participants were divided into the three planned focus groups based on their convenience (i.e., participants’ preference of time and location). One focus group included six participants, and two focus groups included four participants each. All participants were fluent in English and self-reported they were free of neurological and psychiatric conditions [as part of the eligibility screening for the intervention study (Vaportzis et al., 2017b)]. Participants were excluded if they were younger than 65 years or older than 76 years, if they reported conditions that may affect cognitive function (e.g., dementia), and if they were not novice tablet users. Participants also completed the Mini-Mental State Examination (Folstein et al., 1975) as a basic screening for potential cognitive impairment, used for descriptive purposes only. A suggested cut-off point for this test is 26 out of 30, lower scores indicating potential cognitive impairment. All participants obtained scores over 26 as per the recruitment strategy that was to include only cognitively healthy older adults. Demographic information is presented in **Table 1**.

**Table 1:** Demographic characteristics, *N* (%).

	Tablet group		
		
Variable	Focus group participants (*N* = 14)	Total (*N* = 22)	Control group (*N* = 21)
**Sex**			
Female	10 (71.4)	14 (63.6)	15 (71.4)
Male	4 (28.6)	8 (36.4)	6 (28.6)
**Ethnicity**			
White British	13 (92.8)	21 (95.5)	21 (100)
White other	1 (7.2)	1 (4.5)	0 (0.0)
**Education**			
Some high school	1 (7.2)	1 (4.5)	6 (28.6)
High school	2 (14.3)	4 (18.2)	5 (23.8)
Some college	6 (42.8)	9 (40.9)	6 (28.6)
Graduate	3 (21.4)	5 (22.7)	3 (14.3)
Postgraduate	2 (14.3)	3 (13.6)	1 (4.7)
**Living status**			
Alone	7 (50.0)	13 (59.1)	9 (42.9)
Partnered	7 (50.0)	9 (40.9)	12 (51.1)


*Percentages may not total 100% due to rounding.*

### Post-intervention Questionnaire

On the day of post-intervention testing, which was conducted before the focus groups, all participants (i.e., 21 controls and 22 tablet training intervention participants) completed a Post-intervention Questionnaire to give their opinion about tablets and applications (included in **Appendix A**). The questionnaire was created based on previous research, input from colleagues and also our own research interests. Examples of questions that have been previously used in published studies include questions about the perceived ease of use (i.e., I think it is easy to use a tablet) and usefulness of such devices (i.e., A tablet is useful) (Zhou et al., 2014). Based on input from colleagues we added some questions about communication and knowledge sharing. Control participants from the “Tablet for Healthy Ageing” study completed the questionnaire even though they were not subject to the tablet training intervention so that a comparison could be made in the opinions across groups based on their level of exposure. That is, while it would be anticipated that those receiving the intervention would develop a more favorable impression of tablets as a result of their 10-week experience and support within the training classes, that needs to be assessed against those not receiving that engagement. All participants completed the same questionnaire; however, the tablet group was asked some additional questions (e.g., Overall, what is your opinion of the tablet training course?), but apart from those, the questionnaire for the two groups was identical (e.g., How likely it is that you would use a tablet in the future?).

### Focus Groups

Fourteen of the 22 participants who had received tablet training in the “Tablet for Healthy Ageing” study participated in focus groups. We conducted the focus group sessions in February 2016, approximately 2 months after completion of the tablet training course, in a quiet room at Heriot-Watt University, Edinburgh. These focus groups were the last stage of the “Tablet for Healthy Ageing” study and followed a semi-structured agenda with questions informed from previous stages of the study, specifically, the focus groups conducted prior to the intervention phase (Vaportzis et al., 2017a). The focus groups were designed to gain an understanding of older adults’ perception and attitudes toward tablet training following their participation in a tablet intervention. The group discussion lasted approximately 1 h, and the moderator was one of the authors of this study (E.V.). Participants were seated around a table with the moderator being seated with them at the table.

Initially, the moderator reminded participants of the aims of the focus groups and that the discussion would be used to suggest directions for future research. Participants gave written informed consent. The focus groups concentrated on how participants experienced tablet training, what participants particularly enjoyed and what they found challenging about using the tablet, what the perceived advantages and disadvantages of tablet use were, how the use of tablets assisted (or did not assist) in their everyday lives, etc. The specific questions of the semi-structured agenda can be found in **Appendix B**.

All focus group sessions were audio-recorded and later transcribed verbatim. This study was approved by the Heriot-Watt University School of Life Sciences Ethics Committee. All subjects gave written informed consent in accordance with the Declaration of Helsinki.

### Data Analysis

The transcripts from the focus groups were subjected to thematic analysis. Thematic analysis entails the process of encoding qualitative, textual information, and shares in this sense many similarities with content and framework analysis. However, despite the strict procedural nature of coding, and themes that emerge from constant immersion with qualitative data, thematic analysis is more exploratory in nature than content and framework analysis (Joffe and Yardley, 2004). An inductive nature of analysis was maintained throughout, in the sense that there was genuine interest in new themes, potentially different to those that appeared in existing literature. However, it is noted that the researcher’s involvement in the data collection and analysis of previous stages of the study has led to some familiarization with certain concepts (e.g., usability in everyday life) (Boyatzis, 1998). This conceptual organization is reflected in the semi-structured focus group agenda (see **Appendix B**) as a set of underlying ideas which were carefully recorded, and were considered in the later stages of the analysis as themes were finalized.

The structure of the post-intervention questionnaire was analyzed using a principal component analysis. For group comparisons, Mann–Whitney *U*-tests were conducted due to non-random sampling and small sample size. For the focus groups, data analysis was first conducted by one of the researchers (E.V.) and subsequently by an independent researcher with experience in qualitative data analysis to increase confirmability and dependability (M.G.C.). Dependability was met by both researchers keeping a coding manual, which included original extracts from the group discussions and definitions of the emergent themes (Donovan-Hall, 2004; Johnstone, 2006). We carried out inductive thematic analysis using NVivo10 software (QSR International Pty Ltd, 2012). Each of the researchers read the scripts in detail, and then individually coded and categorized data from the same focus group. Data from the other two focus groups were coded by one of the researchers (E.V.), and were reviewed repeatedly with particular attention to refining the codes by both researchers. Through constant comparison, the two researchers captured all diverse views. Constant refining resulted in a list of themes with their importance determined by frequency, but also multiplicity of participants’ views and uniqueness.

## Results

### Post-intervention Questionnaire

The overall experience of the 22 participants who participated in the tablet intervention was positive. On a 7-point Likert scale (1 = Very poor to 7 = Excellent), 9 participants rated their experience “Excellent,” 9 participants rated their experience “Very good,” 3 participants rated their experience “Good,” and one participant rated their experience “Neither good nor bad.” No participants rated their experience “Fair,” “Poor,” or “Very poor,” suggesting that overall, participants enjoyed the tablet course. More than half of the tablet intervention participants (*n* = 12; 54.5%) reported that their communication with other people had improved. In addition, 16 participants (72.7%) said that they shared their knowledge with other people including friends (*n* = 9; 56.3%), family (*n* = 3; 18.7%), friends and family (*n* = 3; 18.7%) and friends, family, and colleagues (*n* = 1; 6.3%). Those who responded positively (*n* = 16, 72.7%) where further asked how they shared their knowledge. One participant said: “I helped a friend to decide to buy a tablet.” Another participant noted: “I showed a friend what you can do on an iPad.” Someone else said: “I explained how the apps I had used worked,” and another participant said: “I described what I had learned and showed on the tablet.” Intervention participants were also asked if their communication had changed as a result of the training. The majority of participants in both groups (tablet intervention participants and controls) reported that it was likely or very likely that they would use a tablet in the future [tablet intervention group, *n* = 20, 95.5%, *M* = 3.7, *SD* = 0.5; control group, *n* = 16, 76.2% *M* = 3.1, *SD* = 1.1; *U*(127.5, *p* = 0.007)].

The majority of participants thought that tablets were useful [tablet group, *n* = 21; 95.5% (*M* = 3.45, *SD* = 0.60), controls, *n* = 18, 85.8% (*M* = 3.10, *SD* = 0.62)], enjoyable [tablet group, *n* = 18, 81.8% (*M* = 3.23, *SD* = 0.75), controls, *n* = 15, 71.4% (*M* = 2.86, *SD* = 0.65)], can make life more comfortable and effective [tablet group, *n* = 17, 77.3% (*M* = 3.09, *SD* = 0.75), controls, *n* = 12, 57.1% (*M* = 2.67, *SD* = 0.79)], and were easy to use [tablet group, *n* = 15, 68.2% (*M* = 2.59, *SD* = 1.14), controls, *n* = 8, 38.1% (*M* = 1.86, *SD* = 0.72)]. Higher mean scores indicate agreement. In addition, most participants in both groups stated that they were interested in using a tablet (tablet group *n* = 20, 91.0%, controls *n* = 17, 80.9%). Despite that, participants had mixed reactions about the ease of learning to use a tablet. In the tablet group, 44.6% (*n* = 12) agreed or strongly agreed that it was easy to learn to use a tablet, while 22.7% (*n* = 5) neither agreed nor disagreed, and 22.7% (*n* = 5) disagreed. In the control group (that is, those who did not participate in the intervention and therefore had no training experience), only 14.3% (*n* = 3) agreed that it would be easy to learn to use a tablet, while 61.9% (*n* = 13) neither agreed nor disagreed and 23.8% (*n* = 5) disagreed or strongly disagreed. When the mean ratings from these items were compared, participants in the tablet training group were significantly more likely to suggest they would use a tablet in the future, that tablets were easy to use and that it was easy to learn to use a tablet; the other responses were not significantly different across groups.

Six questions related to tablet opinion were analyzed using a principal component analysis with Varimax rotation. Two factors explained a total of 80.0% of the variance (Tablet group = 80.8%; Control group = 78.8%). Factor 1 was labeled “Ease of use” and explained 46.9% of the variance (Tablet group = 49.1%; Control group = 43.8%). Factor 2 was labeled “Tablet usefulness” and explained 33.1% of the variance (Tablet group = 31.7%; Control group = 35.0%). The factor loadings are reported in **Table 2**. The PCA results were used to form factor scores based on those items that had loadings above 0.4; we summed the items loading on each of the factors that corresponded to ease of use and tablet usefulness and then we used these composite scores to compare the tablet intervention and controls groups. The tablet intervention group was significantly more likely to state that tablets were easy to use (*M* = 3.56, *SD* = 1.00) compared with the control group [*M* = 2.61, *SD* = 1.01; *U*(130.0), *p* = 0.013]. However, there were no significant group differences in terms of usefulness [Tablet group *M* = 3.28, *SD* = 0.59; Control group *M* = 2.91, *SD* = 0.61; *U*(151.5), *p* = 0.05].

**Table 2:** Summary of the principal component analysis.

Variable	Factor 1 Ease of use	Factor 2 Tablet usefulness
A tablet is enjoyable	0.391	0.784
A tablet is useful	0.214	0.851
A tablet can make life more comfortable and effective	0.029	0.873
I’m interested in using a tablet	0.016	0.824
It is easy to use a tablet	0.956	0.135
It is easy to learn using a tablet	0.934	0.119


### Focus Groups

The analysis of the focus group transcripts revealed participants’ emphasis on the benefits and challenges of the tablet training course as well as the advantages and disadvantages of using tablets. Participants also made suggestions for future studies. We collapsed the codes into three predominant themes: (1) perceptions of tablet training; (2) experience of using tablets; and (3) suggestions for tablet training and other future interventions. Participants’ quotes are presented to illustrate each theme (attributed to participants using the following notation: Group 1, P1 would refer to participant 1 in group 1, for example). A summary of the themes and subthemes is presented in **Table 3**.

**Table 3:** Focus group themes and subthemes.

Theme	Subthemes
(1) Perception of	(a) Confidence
tablet training	(b) Human interaction
	(c) Perceived course outcomes
	(d) Tablet training challenges
(2) Experience of	(a) Advantages of using tablets
using tablets	(b) Potential of tablets to improve older people’s lives
	(c) Disadvantages of using tablets and technologies
	(d) Suggestions for enabling tablet use
(3) Suggestions for	(a) Suggestions for tablet training
future studies	(b) Suggestions for future interventions


#### Perception of Tablet Training

Participants mentioned several benefits as well as challenges of completing the tablet training course. Four subthemes emerged under this theme: (a) confidence, (b) human interaction, (c) perceived course outcomes, and (d) tablet training challenges.

##### Confidence

Thirteen participants mentioned that their confidence increased due to participating in the tablet training course. A participant said: “I do find that I am more confident, more than anything (Group 1, P6).” Participants in Group 3 also felt more confident after participating in the course: “It’s not that I’m afraid to go in to there and look, or anything like that, you know. No fear at all now (P1).”

P4: “She [the instructor] gave us confidence not to be afraid of doing things, yes”

P3: “Yes, I think, as you say, the confidence to do stuff that you like.”

Only one participant mentioned a lack of confidence as a barrier to engaging with their tablet: “I haven’t done Facebook or Skype or anything like that because it was never set up and I don’t have confidence to do that on my own (Group 1, P4).”

##### Human interaction

Eight participants mentioned that they enjoyed the social aspects of the course and being part of a group. One participant noted: “If you’re in a group of people you encourage each other and so you’re getting that sense of wellbeing yourself of doing it, but you can see other people doing it as well (Group 2, P2).” Another participant noted: “And that’s what I enjoyed as well, the group of people. You meet different people from different walks of life (Group 3, P1).” Similarly, another participant said: “Well we had a lot of fun, didn’t we! We had a really fun group and […] that was good (Group 1, P4).”

##### Perceived course outcomes

Discussions also centered around outcomes of the course that participants particularly enjoyed, specifically using the camera and downloading various applications, as the following quotes by Group 1 illustrate:

P3: “I loved it, I loved the photos.”

P4: “Downloading the apps I think was quite helpful. You know, expanding on how much more you can use and get information from, I found, very helpful.”

P1: “We all loved the bus app and [instructor’s name] had funny ones like where’s the nearest public loo!”

##### Tablet training challenges

During the tablet training course, participants sometimes felt challenged as the following quotes by Group 1 suggest:

P1: “I would have liked some of the more basics, rather than just how to use a tablet. You know, the basics of how it actually works because it’s just too modern for my old head.”

P4: “I can learn it, it’s just that it doesn’t come naturally, like with the younger generation.”

P6: “Of course, it’s not at the end of your fingers”.

Keeping up with the expectations of the course was also a challenge sometimes:

P3: “I think the challenge was keeping up to date with what the expectations were for you for each week.”

P1: “I mean it was alright later on because we sort of understood what it was but at the beginning it was like consternation.”

#### Experience of Using Tablets

In addition to the benefits and challenges of tablet training, participants mentioned some specific advantages and disadvantages of using tablets. The four emerging subthemes were: (a) advantages of using tablets, (b) potential for tablets to improve older people’s lives, (c) disadvantages of using tablets and technologies, and (d) suggestions for enabling tablet use.

##### Advantages of using tablets

Advantages of using tablet technology were mentioned. A major advantage was the ability of tablets to allow one to keep in touch with family and friends as Group 1 noted:

P6: “My son is [overseas], so we Skype and on Saturday we were on Skype for an hour and 10 min and he was making his breakfast so I am with him.”

P1: “I’m the same, I’ve got a son [overseas] and I am going to be Skyping him when I get back from here today.”

P3: “Yes, you are never alone. You’re not isolated, you’ve got this piece of equipment which is very modern and up to date. You can contact people. So if there’s an emergency or also other things you can look at if you need to. It can be a lifeline.”

Another advantage of tablets was portability:

Group 2, P2: “So its sheer portability makes it ideal for what it’s designed for, for quick use; you’re not having to open it up, it’s just there ready for you. So its sheer portability I think is its main advantage.”

Group 3, P4 “I had to run up and downstairs between the kitchen and where the computer was before and I have burnt things before. And of course I would have printed it out but – because the tablet is so portable you can take it in to the kitchen, you know.”

##### Potential of tablets to improve older people’s lives

Overall, participants felt that using a tablet could benefit people’s lives. To the question ‘do you think that the tablet improved your mental abilities?’ participants responded:

Group 2, P3: “In general yes, absolutely it does, yeah, because it keeps you thinking, it keeps you active.”

Group 2, P2: “Yes, if you’re using that on a regular daily basis it does improve your brain function.”

Participants from other groups shared similar beliefs:

Group 3, P1: “And there’s some [apps] that make you think fast.”

Group 3, P2: “I found I did get quicker, I did actually notice that.”

Interestingly, the cognitive ability domain that appeared to benefit from the tablet intervention was processing speed (Vaportzis et al., 2017b); neither the moderator nor the focus group participants were aware of this result while the interviews were being conducted. However, one participant noted: “I think I got a bit better at [using the tablet], but otherwise I don’t know (Group 1, P1)”, suggesting that some were skeptical about the potential of tablets to improve mental abilities.

In addition to possible improvements of mental abilities, seven participants also thought that a tablet could potentially have positive effects on other aspects of health and wellbeing. Group 2 for example noted:

P2: “I do personally think, perhaps not for our generation, but certainly from the younger generations who from basically 2 or 3 years old are using this technology now it will help them age healthily, yeah.”

P3: “I think 3 weeks into the course I was taken into hospital and was there for quite some time […] I think I probably have recovered better because of [the tablet] because I’m active again, I’m not sitting in the house dwelling on what might have been or what you know could have been. So yeah probably, and not my physical health so much, as my mental health and therefore it’s made my physical health better.”

##### Disadvantages of using tablets and technologies

Nine participants noted some disadvantages of tablets and technologies, or reservations about their use. Participants’ quotes below reflect a fear of technological addiction.

Group 1, P5: “[…] maybe you would get obsessed with having this piece of technology and become lazy and not do physical things.”

Group 1, P4: “You might lose it. You could become overly obsessed with it. You might put too much in to it. That could be a disadvantage.”

The fear of becoming “addicted” to certain aspects of tablet usage was reflected in the other groups, as illustrated by the following:

Group 3, P2: “It’s quite addictive when you start playing the games […]. Try it again and see if you can do it quicker.”

##### Suggestions for enabling tablet use

During the focus groups, participants mentioned a number of things that might enable tablet use more generally. Seven participants commented on the very small physical features of tablets suggesting that larger buttons might encourage tablet use in this population:

Group 2, P4: “There was an on/off switch, but it was so small and so the lettering was so small, you can’t see it.”

Group 2: P5: “Yes, even with glasses you can’t see it.”

Courses that offer instructions might also encourage tablet use based on participant suggestions: “I think if you buy [brand name] just now they’re running classes (Group 3, P1).” Participants in Group 1 said:

P5: “There are classes, aren’t there? They are silver surfers.”

#### Suggestions for Future Studies

Participants revisited aspects of the course. Two subthemes emerged under this theme: (1) suggestions for tablet training, and (2) suggestions for future interventions in general.

##### Suggestions for tablet training

The tablet intervention classes ran at three different Edinburgh venues. Two of the classes had around 10 participants and one class had around five participants. Participants in the larger classes would have preferred a smaller class:

Group 1, P3: “I would rather have a smaller number.”

Group 1, P1: “I think that you’re right to say that because I am sure that there was a lot of us where our hearing is not as good as it used to be, and certainly with a lot of people it can be very difficult, so that is a problem with older people.”

Participants in another group suggested a smaller tutor/pupil ratio:

Group 2, P4: “I wonder whether the tutor/pupil ratio was right, whether a class of 1:10 or 12 typically at [both venue names], whether that was just slightly too big, that maybe 1:8 might have been more appropriate.”

A participant from the smallest group mentioned: “We were in the group, which was the smallest group, I think, and from what I gather from [instructor’s name] it was probably much easier for her, because we were all talking anyway. I gather that the bigger groups became quite difficult (Group 3, P4).”

Another issue that was raised was the duration of the tablet training course: “I don’t know if the 10 weeks was enough to cover (Group 3, P3)” said a participant. Participants in Group 1 also noted:

P5: “Because people had a lot of questions to ask and maybe the 2 h wasn’t maybe enough.”

P2: “Because maybe some things couldn’t be covered as much as [instructor’s name] would have liked, or as we would have liked.”

Six participants noted that they would have liked a better understanding of the very basic aspects of the tablet.

Group 1, P6: “I think I would have liked, perhaps, to know more about the pad, because if something happened I think ‘Oh heck, what am I going to do here.”’

Group 1, P4: “Especially for me, because I am not computerized or anything like that. I have no phone. The very basics, I mean turning on and off, I get that, but I felt sometimes I made a mistake or I deleted something and I thought ‘What have I done here?’, you know, how do I get it back? Things like that, [instructor’s name] didn’t really cover that.”

Due to library restrictions, WiFi connectivity was not always possible as one participant noted: “[We had] problems with access being in the library (Group 1, P6).” Similarly, someone else mentioned: “The venue probably was the bit that we didn’t like […] because of the connections (Group 3, P3).”

##### Suggestions for future interventions

In addition to feedback specific to the tablet training, we gathered general feedback to guide future research. For example, we asked participants to name things or activities that might be helpful in maintaining or improving mental abilities. Physical activity was one thing that all participants thought might be helpful in maintaining or improving mental abilities. Diet also came up in discussions in Group 1.

P3: “I think exercise.”

P5: “Exercise. Getting myself eating less, you know, and getting out and walking more.”

Participants in Group 3 had a similar discussion.

P4: “Physical activity I’m afraid.”

P3: “The more activity you do the better, really. I think.”

P3: “Yes. Diet and exercise.”

Some participants mentioned dancing, to illustrate their suggestions: “Line dancing is very challenging. But then it’s not everybody that can do it (Group 1, P1).” Other participants mentioned physical activities that encourage mindfulness: “What about Tai Chi, because that seems to be done up to the 90s, you know, these kinds of mindfulness, you know, these kinds of courses I think would be very helpful (Group 1, P3).”

## Discussion

We conducted the current study to gain insights related to older adults’ participation in a tablet intervention program to understand what they found helpful or unhelpful about the tablet training and to guide future research. We employed mixed methods using a questionnaire and post-intervention focus groups to evaluate the tablet training intervention. Participants were invited to share their experiences and insights of engaging with new technology. This approach shows promise for interventions that depend on older adults’ engagement to be effective.

### Post-intervention Questionnaire

As suggested by the post-intervention questionnaire, the majority of participants in the tablet intervention group enjoyed their participation. There was only one participant who rated their experience ‘neither good nor bad.’ This participant had no previous computing experience and felt they would benefit from a more personalized one-to-one course. Overall, both groups emphasized the likelihood of using a tablet in the future. However, the tablet intervention group was significantly more likely to state they were ‘very likely’ to use a tablet compared to the control group. In addition, unlike controls, no participants in the tablet intervention group stated it was ‘unlikely’ or ‘highly unlikely’ that they would use a tablet in the future. The tablet intervention participants were provided with explicit training in how to use a tablet, and as would be expected, reported that they were more likely to adopt this new piece of technology in the future. This may be a result of their increased exposure allowing them opportunities to more accurately determine ease of use and potential utility. This is in line with TAM (Davis et al., 1989) that holds that individuals tend to adopt pieces of technology if they are useful and easy to use. Indeed, we found that two factors explained the overall sample’s responses: the ease of learning to use a tablet, and also the usefulness and enjoyment of it. Although enjoyment is not emphasized by the TAM model, usefulness and enjoyment appear to form a single factor in this study. For example, by enjoying a tablet activity, and therefore being motivated to continue to use it, participants may also appreciate its usefulness. Similarly, by completing useful activities, they may feel enjoyment by the outcome.

### Benefits of Tablet Training

The questionnaire data were reinforced by the focus group findings suggesting that participants found the tablet training course particularly useful for their confidence. Older people may lack confidence when first using tablet (or any other kind of) technology (Czaja and Sharit, 1998). Formal training may introduce technology to older people in an accessible way, and assist them in keeping up-to-date with technological advances and trends (Heinz et al., 2013; Taha et al., 2016). Previous studies have also found that formal computer training of older adults reduced their anxiety and increased their confidence of using technology (Cody et al., 1999). With increased confidence, older people might enjoy the benefits of using new technologies. For example, our results suggested that along with confidence, quick access to information and social inclusion were reported as some of the positive outcomes of the training course. Tablets are less complex than other interfaces (Balagtas-Fernandez et al., 2009) and do not require wired infrastructure. Participants appreciated the quick access to information on the go. In addition, although not exclusive to tablet training, participants enjoyed the social interaction with each other. As people get older and retire, they may miss out on important opportunities for social interaction (Findlay, 2003). The tablet course was a social interaction opportunity for people of similar age and tablet ability.

Despite the positive effects on participants’ overall confidence, some participants mentioned that they did not feel confident enough to complete certain tasks, such as online banking. A recent review (Klimova et al., 2016) suggested that older people use technologies at a basic level, and are quite inefficient at more complex tasks including financial transactions. Our results suggest that positive changes in older adults’ attitudes are possible with appropriate guidance and support. These changes were gradual and required time. Gatti et al. (2017) also found that their tablet program improved older participants’ perceptions in using tablets. They suggested that it is essential that similar programs should follow the learning rhythm of the participants to be effective.

Keeping in touch with family was one benefit of tablet use that our participants noted. This is in line with previous findings. For example, an American Association of Retired Persons [AARP] (2017) survey reported that about 90% of adults over 50 years old use technology to stay connected to family and friends (i.e., 91% 50–59 years, 92% 60–69 years, 88% over 70 years).

### Tablet Training and Mental Abilities

Participants were quite confident that tablet training could have beneficial effects on mental abilities [somewhat consistent with the intervention literature (Chan et al., 2016; Vaportzis et al., 2017b)], and potentially, could improve overall health and wellbeing. Some participants felt that they were quicker due to tablet training as the following quote illustrated: “I found I did get quicker, I did actually notice that.” As noted above, this was particularly interesting as the analysis of the intervention data suggested that participants in the tablet training group had significantly faster processing speed post-intervention compared with controls (Vaportzis et al., 2017b). Therefore, participants not only experienced better processing speed performance, but some of them reported feeling faster versus having better memory or reasoning skills, for example, which were not affected by the intervention.

### Future Research

Our findings may be used to inform future studies implementing interventions (as well as those running training courses with older people out with the context of research). Some suggestions include smaller classes and a longer course duration. Participants in the large classes (10 or more participants) thought they would benefit from a smaller class. Smaller classes might be a particular benefit to those with specific requirements, including hearing problems. Older adults who experience hearing loss have difficulty following conversations in noisy environments or when two or more speakers participate (Murphy et al., 2006). Some participants thought that the duration of the course (i.e., 10 weeks) was not long enough to cover everything. The instructor mentioned that she often had to repeat information, and especially at the beginning of the course, more time than expected was devoted to relatively simple tasks such as turning the tablet on. It is important to repeat instructions to reinforce the information and prevent memory concerns.

Future intervention studies should allow more than a couple of hours for grasping the basics though it is also important to allow learners to participate at their own pace. Our course not only encouraged participants to complete tasks at their own pace and time (e.g., complete homework activities), but also to complete tasks that were of interest to them. Moving at a pace that fails to consider individual needs and abilities can make learning much harder as participants will have to constantly keep up with new challenges before fully mastering earlier skills. Feeling overwhelmed could potentially lead to participants dropping out of studies or classes. Similarly, if participants do not see any utility in the activities they complete and do not enjoy them, the possibility of quitting or not adhering to them is higher.

The study employed a mixed methods design to evaluate the tablet intervention. This is an approach that other researchers might wish to consider when evaluating the impact of interventions, as recommended by the Medical Research Council Framework (Campbell et al., 2000). Issues around standardizing the design and delivery of interventions point to the importance of developing and evaluating interventions, including capturing participants’ views on potential implementation issues such as adherence in the present study, at an earlier stage, and prior to large scale trials (Albright et al., 2013).

### Implications

Implications for tablet and application designers include adaptable font and audio functions. These should allow for high contrast, larger text and louder, clearer sound for those older adults with eyesight and hearing difficulties. Printed instructions on basic tablet functions would also be helpful as some struggled with getting the tablets started. Since we found that tablets have the potential to improve older adults’ mental abilities and possibly overall health, free introductory training courses might encourage older adults to use tablets. Although some brands and some cities (e.g., Edinburgh) offer short tablet courses free of charge, these type of courses should be more widely available and better advertised as some may be unaware of them.

### Future Interventions

Regarding different kinds of interventions that may assist in maintaining or improving cognitive abilities, there was a clear consensus on the potential benefits of physical activity. Some participants mentioned the importance of a healthy diet in conjunction with physical activity suggesting that overall participants were aware of the benefits of engaging in physical activity (Angevaren et al., 2008) and following a healthy diet (Solfrizzi et al., 2003). Applications that encourage or keep track of physical activity and healthy dieting could potentially be beneficial to those who acknowledge the importance of a healthy lifestyle but struggle adhering to it.

### Limitations

One of the limitations of the study is that the majority of participants were female, and therefore, our findings may not translate to males. Nonetheless, the gender imbalance may reflect the trends in the wider society as previous studies found that males are more likely to use or own technological equipment compared with females (Wilson et al., 2003; Pinkard, 2005). One of the criteria for inclusion in the intervention study was to be a tablet novice or have minimal tablet experience. It is likely that fewer males were tablet novices, and therefore, eligible to participate. The sample also comprised a relatively young sample of older adults, and the age range was restricted to control potential cohort effects. Future larger studies may investigate differences in older adults (over 75 years) and between different age groups (e.g., 65–75, 76–85 years, etc.). In addition, the majority of participants were White British, so our sample lacked ethnic diversity. Furthermore, as the sample for all aspects of the study was self-selected, it is important to consider how to ensure the most representative sample is recruited at baseline, and also that participants usually at higher risk of drop-out (for example, due to poorer baseline health, or lower motivation) are retained. The current study cannot specifically address issues of perceptions of technology or adoption in older people with specific impairments for example, which therefore represents an important area for follow-up.

Our questionnaire was not driven by a theoretical framework. However, the focus of the current paper was to explore participants’ experiences of and attitudes towards a tablet training course; therefore, the focus groups represent the main method of collecting that in-depth experiential data. Similarly, the survey questions on ease of use and confidence were only completed post-intervention, and we cannot therefore state that the differences observed were entirely driven by group allocation (though given the randomly allocated groups did not differ at baseline on other substantive factors it would not be expected there would be baseline differences in these measures; future intervention studies related to new skill acquisition should consider this). Therefore, while the quantitative results considered differences between the groups based on their intervention experience, it cannot be stated that these represent a change in their beliefs across time. As the intervention study used a control group that was not involved in any kind of activity, the differences between the two groups may be due to other factors, such as social interaction in the tablet group. Future studies should include a receptive engagement group that participate in a similar degree of social interaction, for example.

## Conclusion

Our findings suggest that the majority of our participants felt that their confidence increased because of participating in the tablet training course. Participants also enjoyed being part of a group highlighting the importance of social interaction in older age. Although participants initially felt out of their comfort zone, upon completion of the course all 14 participants either purchased or intended to purchase a tablet. The majority of participants were confident that tablet training could improve their mental abilities and had the potential to improve other aspects of health and wellbeing as well. Future studies investigating the effects of tablet training on older adults’ cognitive abilities, health and wellbeing are warranted, and should be followed by post-intervention focus groups to deepen our understanding about participants’ intervention experiences.

## Author Contributions

EV conceptualized and designed the study, acquired, analyzed and interpreted the data, and drafted the manuscript. MC analyzed and interpreted the data. AG conceptualized and designed the study. All authors revised the manuscript critically for important intellectual content, gave final approval of the version to be published, and agreed to be accountable for all aspects of the work in ensuring that questions related to the accuracy or integrity of any part of the work are appropriately investigated and resolved.

## Conflict of Interest Statement

The authors declare that the research was conducted in the absence of any commercial or financial relationships that could be construed as a potential conflict of interest.
