# Supplementary material for: Older Adults Experiences of Learning to Use Tablet Computers: A Mixed Methods Study
Source: Front Psychol. 2018 Sep 3;9:1631. doi: 10.3389/fpsyg.2018.01631 (PMC6130193; doi:10.3389/fpsyg.2018.01631)
Supplement: Supplementary file 1 [file Data_Sheet_1.docx]

**Appendix A**

**Tablet Experience Questionnaire (Intervention participants)**

Thank you for participating in focus groups for the study *Tablet for Healthy Ageing.*  Please complete this short survey to give us your honest opinion about your experience with the tablets.

1. **Overall, what is your opinion of the tablet training course?**

- Very poor
- Poor
- Fair
- Neither good nor bad
- Good
- Very good
- Excellent

1. **How likely it is that you would use a tablet in the future?**

- Very unlikely
- Unlikely
- Undecided
- Likely
- Very likely

1. **Has your communication with other people changed as a result of learning how to use a tablet?**

- Yes
- No
- Unsure

If you responded **Yes** to **Question 3, complete Question 3a** and **3b.**

If you responded **No** or **Unsure** to **Question 3,** move to **Question 4.**

**3a. If yes, has it been improved or worsened?**

- Improved
- Worsened

**3b. In what ways has communication been improved/worsened? Please give some examples.**

___________________________________________________________________________________________________________________________________________________________________________________________________________________________________________________________________________________________________________________________________________________________________________________________________________________________________________________________________________________________________________________________________________________________________________________________________________________________

1. **Have you shared the knowledge that you acquired during the tablet course with anyone else?**

- Yes
- No
- Unsure

If you responded **Yes** to **Question 4,** complete **Question 4a** and **4b.**

If you responded **No** or **Unsure** to **Question 4,** move to **Question 5.**

**4a. If yes, who did you share it with?**

______________________________________________________________________________________________________________________________________

**4b. How did you share it?**

_________________________________________________________________________________________________________________________________________________________________________________________________________

1. **How much do you agree with each of the following statements?**

|  | Strongly Disagree | Disagree | Neutral | Agree | Strongly Agree |
| --- | --- | --- | --- | --- | --- |
| I think it is easy to use a tablet |  |  |  |  |  |
| A tablet is useful |  |  |  |  |  |
| A tablet is enjoyable |  |  |  |  |  |
| A tablet can make my life more comfortable and effective |  |  |  |  |  |
| It is easy to learn using a tablet |  |  |  |  |  |
| I’m interested in using a tablet |  |  |  |  |  |

*Questions based on Zhou, Rau and Salvendy [29].

1. **How useful would a tablet be for the following everyday activities?**

|  | Not at all | Not very | Somewhat | Very |
| --- | --- | --- | --- | --- |
| Social networking |  |  |  |  |
| Connecting with friends/family |  |  |  |  |
| Organising daily activities |  |  |  |  |
| Managing and tracking finances |  |  |  |  |
| Health tips and resources |  |  |  |  |
| Exploring family and history |  |  |  |  |
| Sharing photos and memories |  |  |  |  |
| Finding local resources |  |  |  |  |
| Playing games |  |  |  |  |
| Planning a trip or holiday |  |  |  |  |
| Nutrition and finding recipes |  |  |  |  |
| Fitness |  |  |  |  |
| Music |  |  |  |  |
| Watching movies |  |  |  |  |

1. **What other topics might be of interest to you?**

_______________________________________________________________________________________________________________________________________________________________________________________________________________________________________________________________________________________________________________________________________________

**Thank you!**

**Tablet Experience Questionnaire (control participants)**

Thank you for participating in focus groups for the study *Tablet for Healthy Ageing.*  Please complete this short survey to give us your honest opinion about your experience with the tablets.

1. **How likely it is that you would use a tablet in the future?**

- Very unlikely
- Unlikely
- Undecided
- Likely
- Very likely

1. **How much do you agree with each of the following statements?***

|  | Strongly Disagree | Disagree | Neutral | Agree | Strongly Agree |
| --- | --- | --- | --- | --- | --- |
| I think it is easy to use a tablet |  |  |  |  |  |
| A tablet is useful |  |  |  |  |  |
| A tablet is enjoyable |  |  |  |  |  |
| A tablet can make my life more comfortable and effective |  |  |  |  |  |
| It is easy to learn using a tablet |  |  |  |  |  |
| I’m interested in using a tablet |  |  |  |  |  |

*Questions based on Zhou, Rau and Salvendy (Zhou, Rau, & Salvendy, 2014).

1. **How useful would a tablet be for the following everyday activities?**

|  | Not at all | Not very | Somewhat | Very |
| --- | --- | --- | --- | --- |
| Social networking |  |  |  |  |
| Connecting with friends/family |  |  |  |  |
| Organising daily activities |  |  |  |  |
| Managing and tracking finances |  |  |  |  |
| Health tips and resources |  |  |  |  |
| Exploring family and history |  |  |  |  |
| Sharing photos and memories |  |  |  |  |
| Finding local resources |  |  |  |  |
| Playing games |  |  |  |  |
| Planning a trip or holiday |  |  |  |  |
| Nutrition and finding recipes |  |  |  |  |
| Fitness |  |  |  |  |
| Music |  |  |  |  |
| Watching movies |  |  |  |  |

1. **What other topics might be of interest to you?**

_______________________________________________________________________________________________________________________________________________________________________________________________________________________________________________________________________________________________________________________________________________

**Thank you!**

**Appendix B**

**Agenda Questions**

- First, let’s talk about the training overall. Tell me about what you learned from the tablet training course.
- What did you like most about this training course?
- If you were asked to go through this training course again, what are some of the sessions you would wish to visit?
- What are some of things that were challenging to you on this training course? How were you able to overcome these challenges?
- What are the things that you did not like on this training course? What made you not like these things?
- What do you think the advantages of using tablets are?
- What do you think the disadvantages of using tablets are?
- Was the tablet helpful in assisting with everyday living?
- Do you think that the tablet improved your mental abilities? If yes, could you give examples that illustrate this point?
- Do you think that the tablet was helpful in improving your general health and wellbeing? If yes, could you give examples that illustrate this point?
- Can you think of any other things or activities that might be helpful in maintaining or improving mental abilities?

Finally, is there anything else about your experience in the tablet training course that you would like to share?
